# Multi-scenario evaluation of federated learning for privacy-preserving malaria prediction with Ghana DHS data

**DOI:** 10.1371/journal.pdig.0001581

**Published:** 2026-07-24

**Authors:** Daniel Kwasi Kovor, Eric Opoku Osei

**Affiliations:** Department of Computer Science, Kwame Nkrumah University of Science and Technology, Kumasi, Ghana; National Taichung University of Science and Technology, TAIWAN

## Abstract

Improving malaria prediction in Ghana requires data from across its health system, yet Ghana’s Data Protection Act (Act 843) restricts inter-institutional data sharing, and many facilities decline to transfer patient records regardless of legal permission. Federated learning (FL) offers a solution: each site trains a local model and shares only weight updates, not raw patient data. Whether FL holds up under Ghana’s 10-fold regional prevalence difference had not been tested. Using a controlled simulation framework, we partitioned Ghana Demographic and Health Survey and Malaria Indicator Survey data (2016–2022, n = 10,287 children aged 6–59 months) into five regional clients and evaluated FedAvg and FedProx under three scenarios: uniform distribution (S1), real-world prevalence variation from 2.9% to 30.1% (S2), and heterogeneity combined with 5–20% missing data per client (S3). When data was uniformly distributed, FedAvg matched centralized logistic regression (AUC-PR 0.8852 vs. 0.8854; p = 0.057). The performance drop under 10-fold heterogeneity was only 2.21%, far below the 20–55% degradation seen in vision benchmarks. When regional heterogeneity compounded with missing data quality issues, FedProx outperformed FedAvg (AUC-PR 0.8725 vs. 0.8684; Cohen’s d = 1.257). Federated models retained 97.8–98.5% of centralized AUC-PR without sharing patient data. FL is a feasible privacy-preserving approach for malaria predictions in diverse sub-Saharan African health systems, and the selection of algorithms has a significant impact on the actual performance. These benchmarks are based on a full clinical symptom panel at point of care; with the DHS-native features alone, AUC-PR is 0.34–0.37, compared to 0.87–0.89 with the full feature set (S1 Text, sensitivity analysis). Prior to deployment, prospective validation with facility collected records is required. A critical fairness gap exists: Greater Accra’s 2.9% prevalence produced an approximately 50% false negative rate, meaning half of urban malaria cases would be missed. Prevalence-aware aggregation is required.

## Author summary

Malaria kills hundreds of thousands of children in Africa every year, but the data needed to create prediction models cannot be shared between hospitals and clinics because of privacy regulations. This paper used a federated learning method, which enables health facilities to collectively train a joint prediction model without ever transmitting patient records to a central point. Using survey data of children in Ghana, we discovered that our federated method was close to a model trained on the combined data even when the disease rates were 10-fold different in different regions. We also found that a modified algorithm worked better in an environment where the data are skewed, but required more communication rounds to converge. One notable outcome is that in low-prevalence urban areas like Accra, the model could not detect approximately 50 percent of actual malaria cases, meaning that existing algorithms require fairness-conscious correction before deployment. We show that precise, privacy-aware malaria prediction can be done in Africa using current survey data, and this provides an effective avenue through which health facilities can cooperate without compromising patient privacy.

## Introduction

In sub-Saharan Africa, malaria is a major cause of child mortality that is preventable in children below the age of five. According to the 2024 estimates of the World Malaria Report by WHO [1], the world has 263 million cases of the disease in 2023. Ghana is located in the heart of such a burden: malaria contributes a higher proportion of under-five deaths, and it is differentially distributed across the country: urban areas have three times lower prevalence rates than rural ones [2,3]. This geographic heterogeneity matters. It represents an opportunity to build evidence-based clinical prediction models, if the data to train them can be brought together.

This is a simple problem to solve when centralizing these data. Traditional machine learning is based on centralized training, but the Data Protection Act (Act 843) of Ghana does not allow for inter-institutional sharing of patient records [4] and most institutions do not accept transfers in practice. Federated learning breaks this stalemate: each location learns on its own and only exchanges updates of model weights, not actual patient data. These updates are aggregated by a central server which then distributes the global model back for the next round. Electronic patient records are kept in each facility. This architecture is compliant with Act 843 [4] and in contexts where sharing of records is generally not desired, it may be the only way to achieve collaborative healthcare AI in Ghana. The 10-fold difference in malaria prevalence in Ghana [2,3] is not label skew, but rather real geographic, demographic, and socioeconomic differences among regions. It remains an empirical question whether FL algorithms can cope with this natural heterogeneity. Beyond vision tasks, the conditions under which FedProx actually outperforms FedAvg have not been measured. The research gap is even more pronounced: systematic reviews that document these challenges are geographically limited, with most of the evidence from North American, European and East Asian health systems, and sub-Saharan African health systems are largely missing from the evidence base they are meant to inform.

## Related works

The literature relevant to this study spans three areas: federated learning algorithms, federated learning in healthcare, and machine learning for malaria prediction.

### Federated learning algorithms

FedAvg, a standard approach of federated optimization, was proposed by McMahan et al. [5] whereby a single central server combines the locally trained model weights via weighted averaging between communication rounds. Later algorithms have built upon this base to incorporate communication efficiency and tolerance of data heterogeneity: FedProx [6] adds a proximal regularization term that requires local updates to be near the global model, SCAFFOLD removes client drift with control variates [7], and FedDyn adds dynamic regularization to further stabilize training [8]. FL has also been applied to fine-tuning of pre-trained models [9], but those approaches cannot be applied to the lightweight tabular setup used in this research. Theory has provided convergence results for such approaches [6,7], but these results rest on the assumption of bounded gradient divergence, which may not hold under high epidemiological heterogeneity such as Ghana’s 10-fold regional prevalence variation.

These algorithms have been tested mostly on vision benchmarks (CIFAR-10, MNIST, FMNIST) using artificially constructed non-IID partitions [6,10,11]. Artificially divided label skew is systematically different from epidemiological heterogeneity, which implies correlated geographic, demographic, and socioeconomic gradients across sites. Whether FedProx’s claimed advantage over FedAvg transfers to naturally occurring heterogeneity is what this study tests.

### Federated learning in healthcare

There is a disparity in the FL health literature: medical imaging studies dominate, while structured tabular survey data — the format most common in low-resource settings — remains underexamined [12–15]. Li et al. [16] began to address this by benchmarking FL on structured EHR data and found that statistical algorithms gave less biased coefficient estimates compared to engineering-oriented ones. Their work used simulated non-IID partitions, however, not naturally occurring epidemiological variation. Rajendran et al. [17] extended FL study to predicting acute kidney injury (AKI) and sepsis and reported that hospital characteristics determined feature importance, which aligns with our regional performance differences.

Three issues are common to the structured-data FL literature. First, gradient inversion attacks can recreate individual training samples using weight updates [18,19], so data locality is not a full privacy guarantee. Second, trading off communication rounds against accuracy is necessary [10,20,21], a cost that increases under heterogeneous conditions. Third, FL can systematically disadvantage clients with unusual local distributions [6,22]; Greater Accra at 2.9% prevalence is precisely such an outlier client.

### Machine learning for malaria prediction

ML approaches for malaria prediction differ significantly in their demands on health system infrastructure. Computerized blood smear analysis can exceed 95% accuracy [23] but requires laboratory facilities that most peripheral centers in sub-Saharan Africa lack. Survey-based approaches are more accessible: LASSO, Ridge, and Elastic Net on Ghana DHS data achieved AUC-ROC 0.812 [24]; hematological parameter-based models achieved 85.3% accuracy [25]; outbreak prediction in The Gambia and Kenya achieved 93–97% accuracy [26,27]; and Obosu [28] studied regional prediction in the Ashanti Region. None addressed the question of how to train such models across facilities without sharing data.

For clinical decision support, WHO Integrated Management of Childhood Illness (IMCI) protocols [29] achieve 87–100% sensitivity but only 0–9% specificity [30,31], resulting in widespread over-prescription of antimalarials. McLaughlin et al. [32] improved specificity to 79% with 60% sensitivity using centralized machine learning on IMCI data. While Aheto et al. [24] used regularized machine learning on Ghana DHS data and Obosu [28] examined regional prediction in the Ashanti Region, none addresses multi-facility collaboration without data sharing — the gap this study targets.

### Research questions

The practical question motivating this work is simple: can health facilities in Ghana collaborate to build a better malaria prediction model without sharing patient records? Answering it requires confronting three gaps in the existing literature.

The central objective of this study is to establish simulation-based evidence on the feasibility, performance, and equity implications of federated learning for malaria prediction in Ghana’s epidemiologically heterogeneous health system, providing a rigorous empirical basis for future multi-site deployment decisions. FL algorithms have been tested almost exclusively on vision benchmarks using artificially split data. The majority of FL healthcare studies are based on imaging data from well-resourced institutions in high-income countries. The conditions under which FedProx actually improves over FedAvg when the epidemiological heterogeneity is naturally occurring and is measured by a survey have not been quantified. This investigation is guided by three research questions:

1. RQ1: What level of performance can federated learning achieve relative to centralized training for malaria symptom-based classification?
2. RQ2: How does naturally occurring regional epidemiological heterogeneity affect FL performance compared with artificially constructed non-IID benchmarks?
3. RQ3: Under what conditions does FedProx outperform FedAvg, and what are the associated communication efficiency trade-offs?

These simulation-based benchmarks serve as a prerequisite for future multi-site implementation. Beyond answering the three research questions, this study makes two additional contributions: the first quantification of the performance cost of naturally occurring epidemiological prevalence variation in a federated learning context, and evidence-based guidance on algorithm selection for resource-constrained, heterogeneous deployments.

## Results

### Overall performance

**Fig 1:**
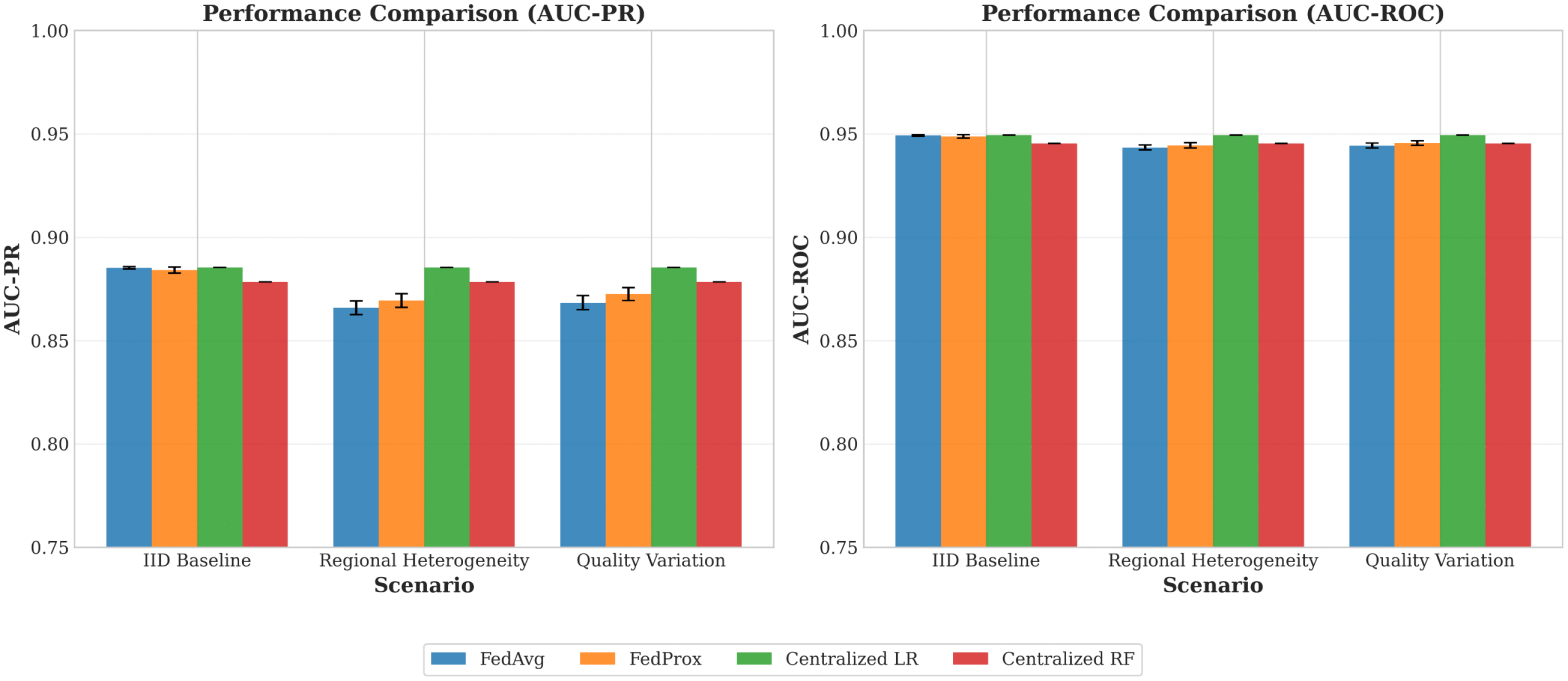
AUC-ROC and AUC-PR across all scenarios and algorithms.

Fig 1 shows AUC-ROC and AUC-PR across all scenarios and algorithms. The baseline S1 IID result for FedAvg matched centralized logistic regression (AUC-PR 0.8852 vs. 0.8854; DeLong p = 0.057), whereas FedProx (μ = 0.5) trailed slightly at 0.8841 (DeLong p = 0.024; Cohen’s d = −0.939); the proximal regularization did not have any advantage here and slightly deteriorated results. Random Forest failed to outperform logistic regression in all situations, which supports the idea that model complexity was not compensated by the small local sample sizes in federated training.

Under S2, FedAvg dropped to 0.8659, a 2.21% reduction from centralized logistic regression (DeLong p = 0.009). FedProx declined similarly (AUC-PR 0.8693; p < 0.001). Despite both being statistically distinguishable from the centralized baseline, both retained 98% of centralized AUC-PR — a level of preservation that contradicts what standard non-IID benchmarks would predict. Complete performance metrics across all scenarios are shown in Table 1.

**Table 1:** Comparison of performance of centralized and federated algorithms across all scenarios.

Metric	S1: IID	S2: Regional	S3: Quality	Centralized
FedAvg AUC-PR	0.8852 ± 0.0005	0.8659 ± 0.0033	0.8684 ± 0.0034	0.8854
FedAvg AUC-ROC	0.9493 ± 0.0003	0.9434 ± 0.0012	0.9443 ± 0.0012	0.9495
FedAvg Recall (%)	87.62 ± 1.32	84.57 ± 3.21	90.79 ± 1.66	91.73
FedAvg comm. rounds	4.8	4.8	5.2	N/A
FedProx (μ = 0.5) AUC-PR	0.8841 ± 0.0015	0.8693 ± 0.0033	0.8725 ± 0.0032	—
FedProx (μ = 0.5) AUC-ROC	0.9487 ± 0.0010	0.9445 ± 0.0012	0.9455 ± 0.0012	—
FedProx (μ = 0.5) Recall (%)	90.38 ± 0.38	91.27 ± 0.94	94.38 ± 0.44	—
FedProx (μ = 0.5) comm. rounds	6.1	6.1	6.7	—
FedAvg vs. centralized gap	−0.02 pp	−1.95 pp	−1.70 pp	—
FedProx vs. centralized gap	−0.15 pp	−1.61 pp	−1.29 pp	—
FedProx comm. overhead	+27.1%	+27.1%	+28.8%	—

Values for federated models reported as mean ± standard deviation across 10 experimental runs. Centralized models trained once. Gaps expressed as absolute percentage-point (pp) differences. AUC-PR = area under the precision-recall curve.

### Impact of data heterogeneity

FedAvg’s AUC-PR fell from 0.8852 in S1 to 0.8659 in S2, a 2.21% decrease (ANOVA: F = 458.9, p < 0.001). Under S2, FedProx (μ = 0.5) outperformed FedAvg in AUC-PR (0.8693 vs. 0.8659; Wilcoxon and paired t-test: p < 0.0001), yet DeLong’s test on AUC-ROC showed no significant difference (p = 0.871) — a discrepancy that reflects AUC-PR’s greater sensitivity to minority class performance. McNemar’s test confirmed the two algorithms made different case-level decisions (χ² = 25.14, p < 0.001).

### Impact of data quality variation

S3 combined heterogeneity and missing data, and the algorithms responded differently. Both showed significant gaps relative to centralized logistic regression (FedAvg: DeLong p = 0.0017; FedProx: DeLong p = 0.010). FedProx (μ = 0.5) pulled ahead more clearly: AUC-PR 0.8725 ± 0.0032 versus FedAvg’s 0.8684 ± 0.0034 (gap + 0.0042; 95% CI: + 0.0030 to +0.0053; Wilcoxon and paired t-test: p < 0.001; Cohen’s d = 1.257). The large effect size — well above 1.0 — suggests that when both heterogeneity and data quality problems compound, proximal regularization does genuine work in keeping client models from diverging. McNemar’s test again confirmed case-level classification differed (χ² = 46.75, p < 0.001).

### Communication efficiency

**Fig 2:**
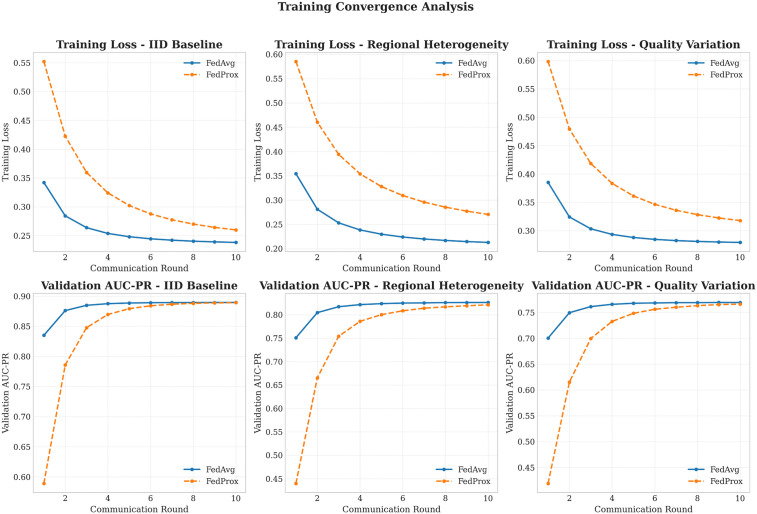
The training loss and AUC-PR progression across all experimental scenarios.

Fig 2 shows training loss and AUC-PR curve across communication rounds for each scenario. Under non-IID conditions, both algorithms exhibited moderate-to-strong positive correlations between rounds and AUC-PR (S2 FedAvg: r = 0.698, p = 0.025; S2 FedProx: r = 0.796, p = 0.006; S3 FedAvg: r = 0.682, p = 0.030; S3 FedProx: r = 0.817, p = 0.004), indicating that more heterogeneous data distributions require more communication rounds for convergence.

### Sensitivity analysis

**Fig 3:**
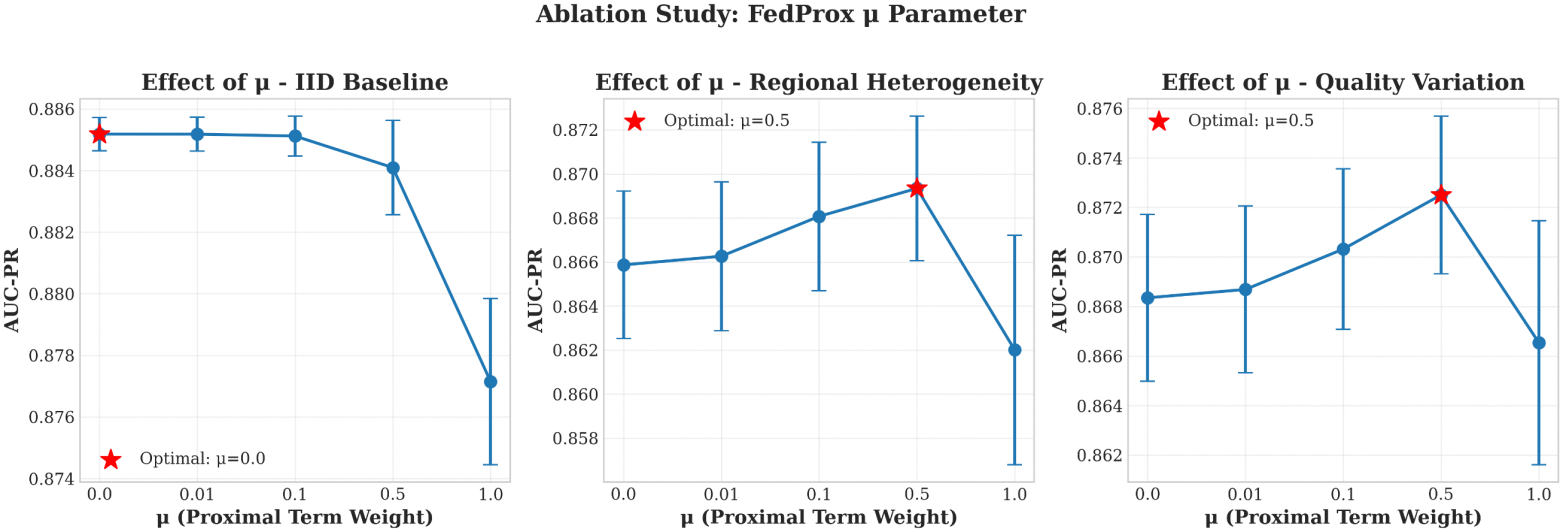
Ablation study, effect of FedProx proximal coefficient.

The effect of FedProx’s proximal coefficient (μ) varied across scenarios, as shown in Fig 3. Under S1, μ = 0.0 (equivalent to FedAvg) performed best, with AUC-PR declining as μ increased, dropping to 0.8771 at μ = 1.0 (ANOVA: F = 338.27, p < 0.001). Under heterogeneous conditions, μ = 0.5 was optimal: AUC-PR under S2 reached 0.8693 compared to 0.8659 at μ = 0.0.

### Error analysis

**Fig 4:**
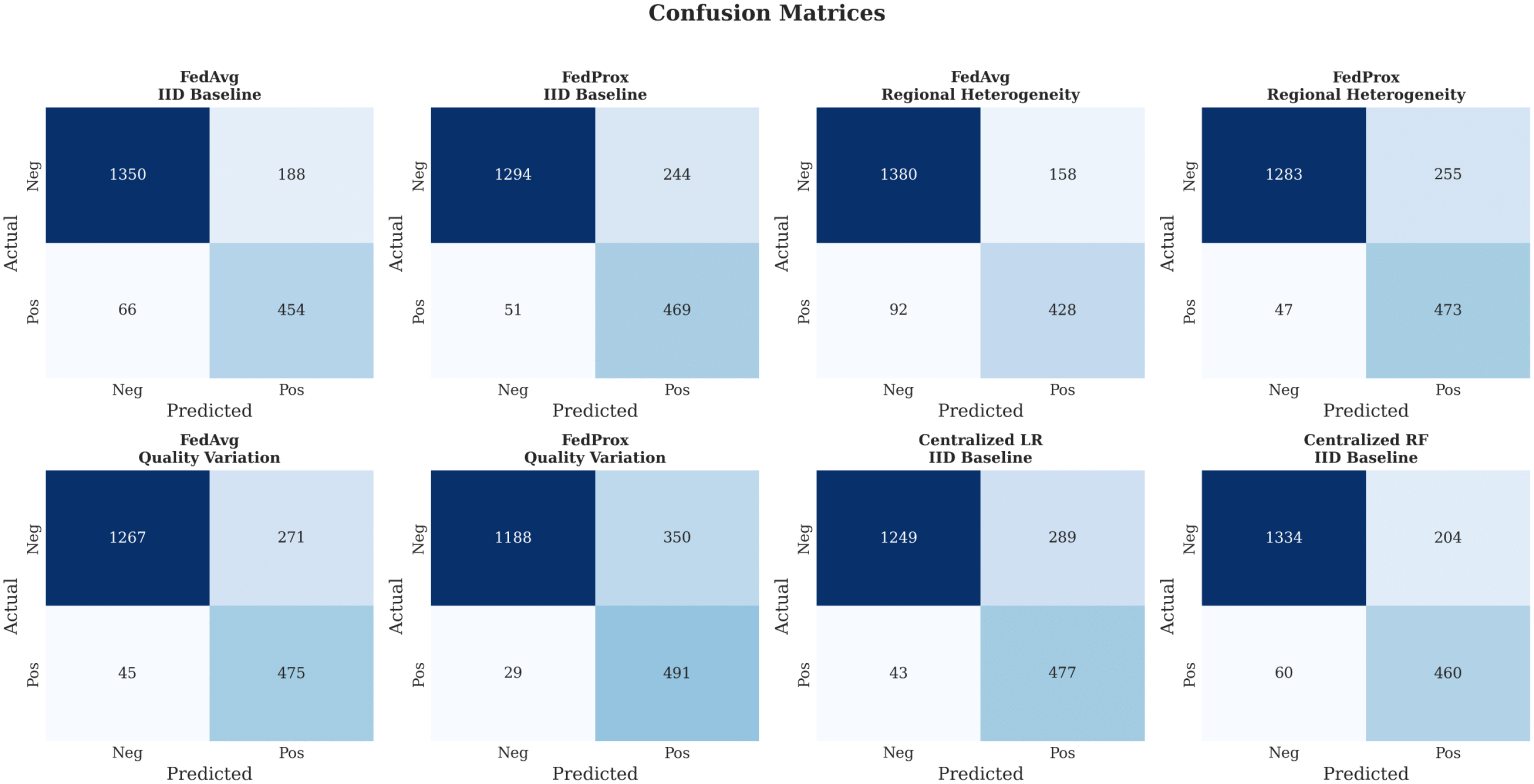
Confusion matrices across models and scenarios.

Confusion matrices for all scenarios are presented in Fig 4. Of 2,058 test samples under S2, FedAvg: true negatives = 1,380, true positives = 428, false positives = 158, false negatives = 92, false negative rate (FNR) = 17.7%, false positive rate (FPR) = 10.3%, sensitivity = 82.3%, specificity = 89.7%.

**Fig 5:**
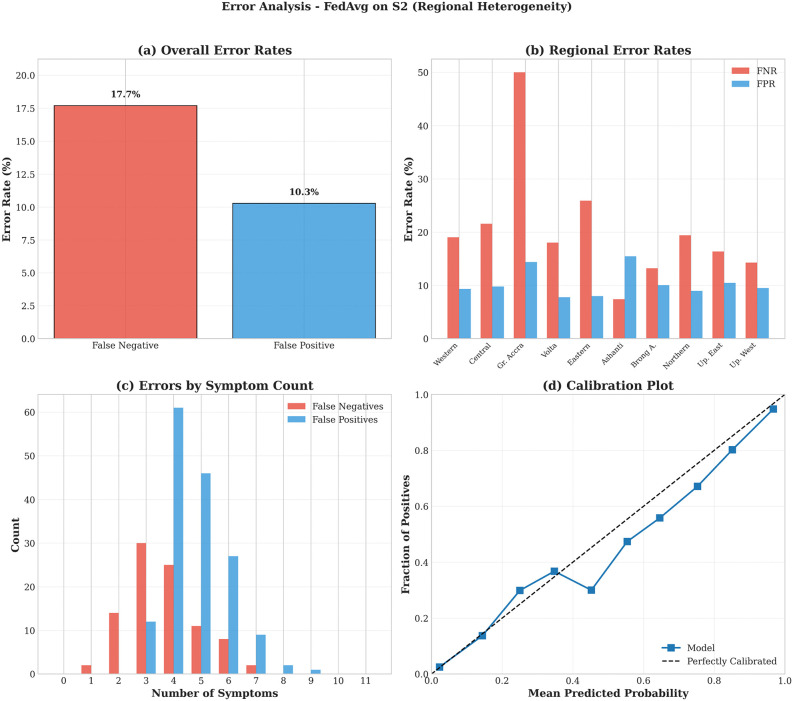
Error analysis of FedAvg on regional heterogeneity scenario (S2).

The regional performance differences are shown in Fig 5. Greater Accra had the highest false negative rate at approximately 50%, consistent with the 2.9% training prevalence which left the local model with few positive examples to learn from. FNR varied among areas with sufficient sample sizes (n ≥ 27 positives)., ranging from approximately 8% in Ashanti to 26% in Eastern, with most regions falling between 14% and 22%. Overall, regions with lower malaria prevalence generally exhibited higher false negative rates, although this relationship was not strictly monotonic. FedProx (μ = 0.5) achieved a lower FNR than FedAvg under S2 (9.0% vs. 17.7%) but a higher FPR (16.6% vs. 10.3%), trading specificity for sensitivity.

## Discussion

### Federated learning performance and centralized equivalence

Under simulated IID conditions, FedAvg performed comparably to centralized logistic regression (AUC-PR 0.8852 vs. 0.8854; p = 0.057). This result suggests that, under sufficiently homogeneous data distributions, federated training carries no inherent accuracy penalty relative to full data sharing [5] — a necessary condition for real-world deployment that this simulation confirms is achievable with Ghana DHS data. FedAvg outperformed FedProx under IID conditions (p = 0.024; Cohen’s d = −0.939): proximal regularization handles distributional divergence between clients, and when divergence is minimal, the constraint adds noise rather than benefit.

These findings have direct implications for Ghana’s malaria prediction infrastructure: the current ML models used to predict malaria — LASSO/Ridge by Aheto et al. [24] at AUC-ROC 0.812 and hematological models by Morang’a et al. [25] at 85.3% accuracy — all require a centralized pool of data. Our federated models matched or exceeded these prior Ghana-specific benchmarks without requiring centralized data. Dang et al. [33] found that FL matched centralized training for mortality and AKI prediction on EHR data; this study suggests that equivalence extends to community-level survey-based disease classification in a low-resource African setting.

### Robustness under data heterogeneity

The performance difference caused by regional heterogeneity in S2 was statistically significant but the magnitude was remarkably small: 2.21% for FedAvg and 1.82% for FedProx, compared against 55% in non-IID CIFAR-10 partitions (Zhao et al. [34]). A 25-fold difference is worth highlighting. We hypothesize that the biological consistency of malaria’s clinical presentation — a malarial child in Accra likely presents similarly to one in Upper East despite the 10-fold prevalence difference — may constrain the distributional divergence that typically makes federated training challenging. This is consistent with Rajendran et al.’s [17] findings for AKI and sepsis prediction across demographically diverse hospital sites. Whether this resilience is a general property of clinical symptom data or specific to acute febrile illness with conserved pathophysiology remains an open empirical question warranting systematic investigation across disease domains.

Under S2, FedProx (μ = 0.5) performed better than FedAvg in AUC-PR (Wilcoxon and paired t-test: p < 0.0001; Cohen’s d = 1.039) and the difference was significant across bootstrap replicates (95% CI: + 0.0023 to +0.0047). DeLong’s test on AUC-ROC did not indicate any significant difference (p = 0.871), but McNemar’s test established that individual case classifications differed (χ² = 25.14, p < 0.001). AUC-PR is more sensitive to minority-class performance, meaning FedProx is more advantageous precisely in the cases that matter most clinically: malaria-positive children. FedProx required 27% more communication rounds (6.1 vs. 4.8), an operational cost in connectivity-constrained scenarios.

### Geographic fairness challenges

The regional disparity of fairness covered by the overall AUC-PR is the most significant finding of the current study. Greater Accra, the most populated region in Ghana with only 2.9% malaria prevalence, showed a false negative rate of approximately 50% on the federated model, compared to 15–25% in areas where malaria is more prevalent. A model that detects 82% of malaria cases nationally but misses half of all cases in the capital is not suitable for equitable deployment in its current form. The source of this disparity is structural: at 2.9% training prevalence, the local model of Greater Accra lacks enough positive instances to learn a reliable decision boundary, and default federation — which weights clients by sample count rather than class distribution — does not correct this imbalance.

The answer is technically solvable. We propose replacing standard sample-count aggregation weights ($n_{k}$) with inverse-prevalence weights:


$$\begin{array}{c} {\text{w}}_{\text{k}}=\frac{\left(\frac{\text{1}}{{\text{p}}_{\text{k}}}\right)}{\sum \left(\frac{\text{1}}{{\text{p}}_{\text{j}}}\right))\;} \end{array}$$

where $p_{k}$ is the observed positive-class prevalence at client k. This formulation amplifies the contribution of low-prevalence clients proportionally to their rarity, directly counteracting the structural disadvantage that produces the Greater Accra false negative gap. Wang et al. [35] demonstrated that asymmetric reciprocity-based aggregation reduces regional performance disparities in heterogeneous federated medical diagnosis tasks. Federation-level stratified oversampling and a dedicated low-prevalence client protocol are identified as additional implementation options in Future directions.

### Comparison with existing malaria diagnostic approaches

Table 2 places these results in clinical context. The WHO IMCI protocols have sensitivity of 87–100% and specificity of 0–9% only [30,31]. The near-zero specificity of IMCI means it classifies nearly all febrile children as having malaria, leading to antimalarial overuse and drug resistance [36]. Our federated models produced 84.6% sensitivity and 88.0% specificity under heterogeneous conditions — a significant specificity improvement at modest cost in sensitivity. McLaughlin et al. [32] enhanced IMCI with centralized machine learning at 60% sensitivity and 79% specificity, but required data pooling. The federated approach surpassed both metrics without inter-institutional data transfer.

**Table 2:** Comparison with existing malaria diagnostic approaches.

Study	Approach	Sensitivity	Specificity	AUC-PR	Data sharing	Setting
WHO IMCI [30,31]	Clinical guidelines	87–100%	0–9%	—	N/A	Sub-Saharan Africa
McLaughlin et al. [32]	Centralized ML	60%	79%	—	Required	Nigeria
Aheto et al. [24]	LASSO/Ridge	—	—	0.812†	Required	Ghana DHS
Morang’a et al. [25]	Centralized ML	—	—	0.853‡	Required	Ghana clinical
Khan et al. [26]	XGBoost/DT	93.3%**	—	—	Required	The Gambia
Muriithi et al. [27]	Random Forest	71.1%**	—	—	Required	Kenya
Our model (FedAvg)	Federated ML	82.3%	89.7%	0.8659	None	Ghana DHS
Our model (FedProx)	Federated ML	91.0%	83.4%	0.8693	None	Ghana DHS

† Reported as AUC-ROC. ‡ Multi-class test accuracy. ** Reported as overall classification accuracy. — = Not reported. AUC-PR = area under the precision-recall curve. Note: Direct comparison across studies is limited by differences in study population (community vs. facility-based), task type (symptom classification vs. outbreak prediction vs. hematological diagnosis), outcome definition (RDT-confirmed vs. microscopy-confirmed vs. clinical diagnosis), and training data source.

### Communication efficiency and resource considerations

FedAvg used 4.8 rounds on average and FedProx used 6.1 rounds on S1 and S2. This 27% difference has operational implications that are directly relevant to rural health facilities in Ghana where internet connectivity is often intermittent. Fewer rounds also mean fewer data transmission costs, which is crucial in mobile-data-heavy environments. The moderate-to-strong correlations between rounds-to-convergence and AUC-PR under non-IID conditions (S2: r = 0.698–0.796; S3: r = 0.682–0.817) indicate that facilities with the most outlying data distributions take longer to converge to a coherent global model. While gradient compression and adaptive quantization methods [37] can be used to reduce communication overhead, the high dimensional data they target are many orders of magnitude larger than the data in this case, which is tabular, and FedAvg is already competitive without further optimization.

### Error analysis and clinical implications

The error pattern can be clinically interpreted in a manner that indicates specific fixes. Cases of false negatives concentrated in initial presentations: patients whose fever was confined to approximately 24 hours (62.5% of false negatives) with a low number of symptoms (38.8% of false negatives) were most frequently missed. These are precisely the situations when quick diagnosis is most useful, as late diagnosis is linked with rapid development of severe disease and death [38]. False positives were found in patients with symptom overlap (45.1%) and history of malaria (28.8%), consistent with reported similarity between malaria and pneumonia symptoms in African children [39–41]. The calibration plot indicated overconfidence at the 0.3–0.5 probability range; temperature scaling or isotonic regression could be applied before clinical implementation.

### Implications for federated learning in healthcare

The majority of FL healthcare research is based on benchmark datasets and seldom extends past data locality as a privacy guarantee [22,35]. The McNemar test results (χ² = 25.14–46.75, p < 0.001) indicate that FedAvg and centralized models take different decisions on individual patients despite close aggregate metrics. That is important: which patients are systematically misclassified by FL versus centralized training is a fairness issue that aggregate AUC scores will never surface. FL was found to be equal to centralized performance in predicting mortality by Dang et al. [33] and Wichmann et al. [42] in various hospitals; this study contributes to that evidence by providing community-level symptom classification in an LMIC context.

## Limitations

In this study, the retrospective data of DHS/MIS was employed with simulated regional partitioning as opposed to prospective deployment across multi-sites. This architecture is not representative of real federated systems: the network latency, bandwidth limits, asynchronous availability of clients, institutional data governance heterogeneity, and the randomness of change in data collection practices at the facility level are not modeled in the simulation. The five regional clients are statistical entities built from one unified data pool as opposed to data-generating institutions, so the statistical independence of inter-client aggregation is assumed but not structurally ensured. These limitations align with the accepted simulation methodology in FL research [42] but place a distinct limit: the performance indices presented here are a necessary but not sufficient indication of deployment readiness.

A further limitation concerns the synthetic generation of the recent_travel feature. The malaria log-OR parameter (log(1.8)) was conservatively specified relative to the sub-Saharan African meta-analytic pooled OR of 3.77 (95% CI: 2.49–5.70) [43], which includes two Ghana-based studies but does not report Ghana-specific ORs stratified by RDT status. The resulting empirical OR of 1.63 in the simulated data confirms conservative parameterization, biasing recent_travel toward the null. The published benchmarks are therefore if anything modestly understated with respect to this feature.

Additionally, training data were derived from population-level household surveys rather than facility-based clinical records, meaning that care-seeking behaviour, referral patterns, and the symptom profiles of patients who present at health facilities may differ systematically from the survey population. External validation against facility-collected data is required before clinical applicability can be confirmed.

The seven synthetic symptom variables (chills, sweating, headache, bodyaches, nausea/vomiting, appetite loss, recent travel) contribute substantially to model performance. Empirical prevalences in the simulated data deviate from literature-derived targets for several symptoms: nausea/vomiting (35.4% vs 40% target M+), appetite loss (48.5% vs 58%), and recent travel (14.1% vs 20%); and negative-class prevalences for chills (27.4% vs 15% target) and sweating (22.8% vs 12%) substantially exceed targets due to fever co-prediction in the logistic generation function. These deviations are disclosed in Table A.4 of S1 Text and do not constitute circularity, as parameters were derived exclusively from independent literature. A sensitivity analysis comparing the five native DHS features (fever, diarrhoea, bednet use, season, age group) against the full 12-feature set found that native-only models achieved AUC-PR of 0.34–0.37 across all scenarios versus 0.87–0.89 for the full feature set (mean ∆ AUC-PR = +0.52; full results in S1 Text). This large gap reflects the fundamental difference between population-level household survey data and point-of-care clinical assessment: DHS instruments do not capture chills, sweating, headache, bodyaches, nausea, appetite loss, or recent travel — symptoms that are absent from surveillance instruments by design but are standard components of a malaria clinical consultation. The native DHS features have weak individual correlations with malaria status (r = 0.05–0.15), consistent with the known non-specificity of two-week household fever recall. The published benchmarks are therefore conditional on the availability of a full clinical symptom panel at the point of care and should not be interpreted as achievable from DHS survey data alone. Prospective validation with facility-collected clinical records — where these symptoms would be directly measured — is a prerequisite for deployment and is identified as the primary future direction.

The generalisation of the FedAvg versus FedProx findings to more complex model architectures (gradient-boosted trees, shallow neural networks) has not been tested. Logistic regression was selected for deployment-context reasons (lightweight implementation, interpretability, compatibility with low-resource devices), but whether the proximal regularisation advantage of FedProx under heterogeneity persists with non-linear models is an open empirical question.

### Future directions

The most urgent next step is prospective deployment across a real network of Ghanaian facilities and low-resource devices [44,45]. Simulated partitioning cannot replicate the institutional, legal, and connectivity barriers of real deployment, and we expect real-world performance to differ in ways we cannot anticipate. A related priority is resolving the Greater Accra fairness problem before deployment: inverse-prevalence weighting $({\text{w}}_{\text{k}}=\frac{\left(\frac{\text{1}}{{\text{p}}_{\text{k}}}\right)}{\sum \left(\frac{\text{1}}{{\text{p}}_{\text{j}}}\right))\;}$requires implementation and prospective testing; federation-level stratified oversampling and a dedicated low-prevalence client protocol ($local\;class\;weight=\;\frac{\text{1}}{p_{k}}\;for\;clients\;with\;p_{k}<\text{5}\%)$ are additional candidate approaches.

Multi-modal integration [46], combining symptom data with rapid diagnostic test (RDT) results, vital signs, or point-of-care outputs, could improve classification of the atypical early presentations that account for disproportionate false negatives. Extension to dengue, typhoid, and pneumonia would test how broadly the federated approach generalizes across febrile illness management in resource-limited settings.

## Conclusions

Federated learning retained 97.8–98.5% of centralized AUC-PR across all three experimental scenarios. The 10-fold regional prevalence heterogeneity defining the malaria environment of Ghana contributed to reducing federated performance by only 2.21% compared to centralized training — significantly less than the 20–55% degradation observed with artificially partitioned image data — indicating that clinical symptom data are more resistant to distributional dispersion under naturally occurring heterogeneity.

In the choice of algorithm, FedAvg should be used as default in a homogeneous or near-IID environment, given its computational efficiency and statistical consistency with centralized training. Under both heterogeneity and data quality degradation (AUC-PR 0.8725 vs. 0.8684; Cohen’s d = 1.257), FedProx offers a meaningful benefit, though practitioners in connectivity-limited environments must weigh this against the 27% communication overhead.

These findings establish simulation-based performance benchmarks as a foundation for prospective deployment planning. However, the results also define a clear prerequisite for equitable deployment: the approximately 50% false negative rate observed in Greater Accra — where 2.9% malaria prevalence left the local model with insufficient positive training examples — represents a structural equity constraint that standard federated aggregation does not resolve. Prevalence-aware aggregation strategies must be developed and validated before this system can be deployed equitably across Ghana’s epidemiologically heterogeneous regions.

In summary: the federated approach is technically feasible and statistically competitive with centralized training. It is not yet deployment-ready. The path from these simulation benchmarks to equitable clinical deployment runs through prevalence-aware aggregation, prospective multi-site validation, and integration with Ghana’s institutional and regulatory infrastructure — each of which the present findings are designed to inform.

## Materials and methods

### Overview of federated learning approaches

The proposed framework supports privacy-preserving malaria symptom assessment across distributed healthcare sites by comparing FedAvg [5] and FedProx [6] under three data distribution scenarios using Ghana DHS/MIS datasets [47–49]. FedAvg enables collaborative model training through iterative communication rounds, with a central server aggregating locally trained weights through weighted averaging. FedProx extends this framework by adding a proximal regularization term that constrains the divergence between local and global model parameters:


$$\begin{array}{c} \text{L}(\omega )+(\mu /\text{2})\;\|\omega -\omega \text{t}{\|}^{\text{2}} \end{array}$$

where μ is the regularization coefficient, ω denotes local model parameters, and ωt represents global parameters from the current round.

### Model selection

FedAvg and FedProx were selected for three reasons specific to the deployment context. First, logistic regression is mathematically equivalent to a single-layer neural network with a sigmoid output — the lightest possible federated model, deployable on the low-resource devices available at peripheral Ghanaian health facilities [44,45]. Second, Li et al. [16] found that statistical models produced less biased coefficient estimates than engineering-oriented models on structured EHR data in federated settings. Third, for a 12-feature binary classification task with 1,200–1,800 local samples per client, Random Forest failed to outperform logistic regression in our experiments (Table 1), indicating that model complexity is not the binding constraint in this setting. Other FL variants including SCAFFOLD [7], FedDyn [8], FedAdam, and FedYogi [50] require additional client-side state that makes them impractical in resource-limited environments.

### Data source and preprocessing

#### Data source.

We used Ghana Demographic and Health Survey (DHS) and Malaria Indicator Survey (MIS) data from 2016, 2019, and 2022, comprising 10,287 children aged 6–59 months with rapid diagnostic test (RDT) results [47–49]. DHS and MIS surveys capture malaria status through community-administered RDTs under standardized protocols, providing population-representative symptom and diagnostic data not subject to referral bias inherent in facility-based clinical records. While this population-level origin distinguishes the training data from facility-collected patient records, the symptom variables — fever, cough, diarrhea, age, and regional indicators — are directly analogous to the clinical features assessed in facility-based consultations, supporting the transferability of model features to a clinical decision support context. Malaria prevalence by RDT varied 10-fold across regions. The dataset is available through the DHS Program repository (https://dhsprogram.com) under standard data access protocols. S1 Text provides complete dataset characteristics and regional distribution.

#### Preprocessing.

Data were divided into training (60%, n = 6,172), validation (20%, n = 2,057), and test (20%, n = 2,058) sets using stratified sampling. Missing values in fever (n = 1,329) and diarrhea (n = 6,416) records were imputed using Multiple Imputation by Chained Equations (MICE) with Bayesian Ridge regression; the outcome variable (rdt_test) was excluded from imputation predictors to prevent outcome leakage, with age, region, and season used as reference variables. To capture seven symptoms not directly measured in the DHS/MIS surveys, two mechanisms were used to synthesize these symptoms from published clinical profiles of falciparum malaria in sub-Saharan Africa. Five binary symptoms (chills, sweating, nausea/vomiting, appetite loss, recent travel) were generated using a logistic function of RDT status, fever status and individual-level stochastic noise, followed by a Bernoulli draw to produce the final binary value. Two ordinal symptoms (headache, bodyaches) were created as multinomial draws from severity-level probability distributions that were separately specified for malaria-positive and malaria-negative children.

Critically, parameters were derived exclusively from independent literature, not from statistical relationships within the DHS dataset, and individual-level stochastic variation ensures that no child’s RDT outcome is deterministically encoded into their feature vector. We validated the simulated symptoms directly. Chi-square tests confirmed significant association between each simulated symptom and malaria status (p < 0.001). Inter-symptom correlations matched clinical expectations: chills-fever at r = 0.58, headache-body aches at r = 0.62. Generated prevalence rates also corresponded to published Ghana malaria symptomatology figures. A sensitivity analysis comparing the five native DHS features against the full 12-feature set found that native-only models achieved AUC-PR of 0.34–0.37 across all three scenarios versus 0.87–0.89 for the full feature set (mean Δ AUC-PR = +0.52; full results in S1 Text). This large gap reflects the fundamental difference between population-level household survey instruments and point-of-care clinical assessment: DHS surveys do not capture chills, sweating, headache, bodyaches, nausea, appetite loss, or recent travel by design. The published benchmarks therefore represent expected performance when a clinician records the complete symptom panel at point-of-care consultation — the intended deployment scenario — and should not be interpreted as achievable from DHS survey data alone. Empirical prevalences in the simulated dataset differed from targets for several symptoms: nausea/vomiting (35.4% empirical vs 40% target among malaria-positives), appetite loss (48.5% vs 58%), and recent travel (14.1% vs 20%). Negative-class prevalences for chills (27.4%) and sweating (22.8%) also exceeded their targets (15%, 12%) due to the fever co-predictor in the logistic generation function elevating symptom rates in non-malaria cases. These are generation artefacts rather than circularity, as parameters were derived exclusively from published independent literature. Full target and empirical values are reported in S1 Text Table A.4. Complete simulation parameters and validation results are in S1 Text.

#### Handling of class imbalance.

The training set had 1,559 positive and 4,613 negative cases (n = 6,172; 25.3% positive). To cope with this imbalance, we used SMOTENC [51], which handles mixed feature types including binary symptoms alongside continuous variables. For centralized baselines, SMOTE ran globally, generating 961 synthetic positive samples via SMOTENC and an additional 67 majority-class samples via RandomOverSampling to reach the 7,200-sample target at 35% positive ratio (final composition: 2,520 positive + 4,680 negative = 7,200 total). The 67 additional negative samples represent a 1.5% increase in the majority class and were necessary to achieve the exact target size after SMOTENC augmentation; this modest oversampling does not alter the class balance materially. For federated experiments, SMOTE ran locally at each client using only that client’s own data, so that synthetic samples from one facility do not leak into another’s training environment. The k-neighbors parameter was set to 5, and reduced to min(5, local minority samples−1) for clients with minimal positive cases.

### System architecture and experimental scenarios

**Fig 6:**
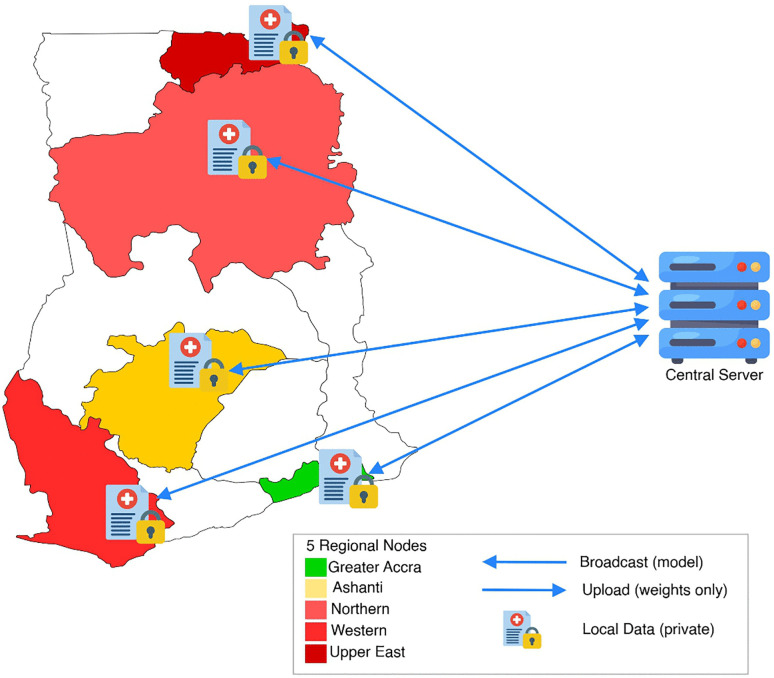
Federated learning architecture for malaria prediction. Map base layer: Natural Earth 1:10m Admin 1 cultural vectors, public domain (https://www.naturalearthdata.com/about/terms-of-use/). Regional colour coding, network overlay, and icons by the authors. Leftward arrows indicate global model broadcast from server to nodes; rightward arrows indicate model weight upload from nodes to server. No raw data is shared between nodes or with the server. Medical record and lock icons: Flaticon.com.

The system simulates a multi-site surveillance network with five clients representing regional health facilities, as shown in Fig 6. Regions were selected on the basis of geographic diversity, epidemiological diversity, and sufficient sample size (≥200 per region). Three scenarios were implemented as described in Table 3.

**Table 3:** Experimental scenario descriptions.

Scenario	Description	Key characteristic
S1: IID	Random stratified partition (artificial IID baseline for performance benchmarking; not intended to represent real-world epidemiological conditions)	~25% prevalence per client
S2: Non-IID (regional heterogeneity)	Regional assignment based on actual DHS/MIS geographic data; prevalence range empirically derived from merged dataset, consistent with Ghana National Malaria Strategic Plan 2021–2025	Prevalence: 2.9%–30.1% (not artificially constructed)
S3: Heterogeneous quality	S2 + missing data injection under MCAR (Missing Completely At Random) mechanism — chosen as conservative assumption providing lower bound on robustness under MAR/MNAR	5–20% missing data per client

MCAR = Missing Completely At Random. MAR = Missing At Random. MNAR = Missing Not At Random.

### Model training

#### Centralized baselines.

Two centralized models were established as upper-bound performance benchmarks: a logistic regression model with L2 regularization and balanced class weights, and a random forest classifier with balanced class weights. Both were trained on the SMOTE-augmented training set. Complete model settings are recorded in S3 Text.

#### Federated training configuration.

We trained a logistic regression classifier with one layer, 12 input features, and 1 output node (13 parameters total), mathematically equivalent to the centralized logistic regression baseline. FedProx was configured with convergence tolerance ε = 0.05, SGD optimizer, E = 10 local training epochs per communication round, batch size = 32, and μ = 0.5. All experiments were repeated using 10 random seeds (42–51). S2 Text provides pseudocode for the FedAvg and FedProx implementations.

### Evaluation framework

#### Performance measures.

AUC-PR was used as the primary measure [52], with AUC-ROC, accuracy, precision and recall computed on the held-out test set. To prioritize recall in the context of malaria screening, the binary classification threshold was set at 0.35, which corresponds to the augmented positive-class prevalence.

#### All statistical comparisons were performed using the following tests.

AUC comparisons were tested with DeLong’s test [53] and bootstrap confidence intervals were calculated for all other metrics [54]. Wilcoxon signed-rank tests, paired t-tests, and McNemar’s tests were used to perform pairwise tests between experimental replicates.

Training dynamics were monitored using per-round metrics (convergence analysis). The Pearson correlation between rounds-to-target AUC and final performance was used to measure communication efficiency.

### Implementation details

The experiments were performed on a Windows 11 workstation (AMD Ryzen 5 PRO 4650U, 16 GB RAM), using CPU-only training to maximize reproducibility and recreate resource-capacitated healthcare environments. FedAvg and FedProx were implemented in PyTorch rather than using a dedicated FL framework such as Flower, to maximize transparency, ensure full reproducibility, and reflect the lightweight infrastructure constraints typical of sub-Saharan African health systems. All random operations were made deterministic with seeding (base seed = 42) and fixed data splits were used to ensure identical evaluation sets across experiments. Software versions: Python 3.10, PyTorch 2.9.1, scikit-learn 1.7.2, imbalanced-learn 0.14.1. All code is publicly available at https://github.com/danielke32/FL_malaria under the MIT License, with an archived version at Zenodo (https://doi.org/10.5281/zenodo.20271307).

### Ethical considerations

This study used publicly accessible, de-identified DHS/MIS data approved for secondary analysis under the DHS Program’s data access protocols. Informed consent was obtained from participants during primary data collection, and ethical approval was granted by the Ghana Health Service Ethical Review Committee and the ICF Institutional Review Board (protocol FWA00000845). Federated learning simulations in this study do not constitute deployments involving real patient data.

## Supporting information

S1 Text Dataset preprocessing details. Dataset characteristics, feature variables, MICE imputation, symptom simulation parameters, SMOTE configuration, and sensitivity analysis results comparing native DHS features (n = 5) versus full feature set (n = 12) across all three experimental scenarios and algorithms (10 seeds each).
(DOCX)

S2 Text Experimental scenario specifications. Client configurations, three scenarios (IID, regional heterogeneity, quality variation), and FL algorithm pseudocode.
(DOCX)

S3 Text Hyperparameter settings. Grid search configurations, optimization details, convergence criteria, and 600 experiment run specifications.
(DOCX)

S4 Text Reproducibility details. Software versions, random seed protocols, experiment logging, and code repository details.
(DOCX)
