# Supplementary material for: Multi-scenario evaluation of federated learning for privacy-preserving malaria prediction with Ghana DHS data
Source: PLOS Digit Health. 2026 Jul 24;5(7):e0001581. doi: 10.1371/journal.pdig.0001581 (PMC13399501; doi:10.1371/journal.pdig.0001581)
Supplement: S1 Text — Dataset characteristics, feature variables, MICE imputation, symptom simulation parameters, SMOTE configuration, and sensitivity analysis results comparing native DHS features (n = 5) versus full feature set (n = 12) across all three experimental scenarios and algorithms (10 seeds each). (DOCX) [file pdig.0001581.s001.docx]

**S1 Text. Data Preprocessing Details**

Comprehensive documentation of data source characteristics, feature variables, missing data imputation procedures, symptom simulation parameters and validation, data splitting configurations, and sensitivity analysis results for the federated learning malaria prediction study.

# A.1 Data Source and Selection Criteria

Data were extracted from Ghana Demographic and Health Surveys (DHS) and Malaria Indicator Surveys (MIS) for 2016, 2019, and 2022. The surveys employed two-stage stratified cluster sampling with enumeration areas as primary sampling units and households as secondary units.

**Table A.1: Dataset Characteristics by Survey Year**

| **Characteristic** | **2016 MIS** | **2019 MIS** | **2022 DHS** | **Combined** |
| --- | --- | --- | --- | --- |
| Total children (6–59 months) | 2,892 | 3,245 | 4,150 | 10,287 |
| RDT positive (%) | 27.8% | 21.4% | 16.9% | 21.2% |
| Regions covered | 10 | 10 | 16→10* | 10 |
| Urban (%) | 42.1% | 44.3% | 46.8% | 44.6% |
| Rainy season (%) | 58.2% | 61.4% | 55.7% | 58.1% |

**2022 regions harmonized to 10-region system: Western North→Western; Ahafo, Bono, Bono East→Brong Ahafo; Oti→Volta; Savannah, North East→Northern.*

## Inclusion and Exclusion Criteria

**Inclusion criteria:** Children aged 6–59 months; complete RDT results; valid demographic variables; selected for biomarker module (HV042=1); slept in household previous night (HV103=1).

**Exclusion criteria:** Missing/invalid RDT results; age outside 6–59 months; missing region or residence information.

# A.2 Feature Variables

**Table A.2: Feature Variables Used in Model Training**

| **Variable** | **Type** | **Source** | **Description** |
| --- | --- | --- | --- |
| fever | Binary | DHS/MIS | Fever in last 2 weeks (0/1) |
| diarrhea | Binary | DHS 2022 | Diarrhea in last 2 weeks (0/1) |
| chills | Binary | Simulated | Presence of chills (0/1) |
| sweating | Binary | Simulated | Excessive sweating (0/1) |
| headache | Ordinal | Simulated | Headache severity (0–3 scale) |
| bodyaches | Ordinal | Simulated | Body aches severity (0–3 scale) |
| nausea_vomiting | Binary | Simulated | Nausea or vomiting (0/1) |
| appetite_loss | Binary | Simulated | Loss of appetite (0/1) |
| bednet_use | Binary | DHS/MIS | Slept under ITN previous night (0/1) |
| recent_travel | Binary | Simulated | Travel in last 2 weeks (0/1) |
| season | Binary | Derived | Rainy (Apr–Oct) vs dry (Nov–Mar) |
| age_group | Categorical | DHS/MIS | 5 categories: 6–11, 12–23, 24–35, 36–47, 48–59 months |
| malaria_status | Binary | DHS/MIS | Target: RDT result (0=negative, 1=positive) |

# A.3 Missing Data Imputation (MICE)

**Table A.3: Missing Data Patterns and Imputation Results**

| **Variable** | **Missing (n)** | **Missing (%)** | **Imputation Method** |
| --- | --- | --- | --- |
| fever | 1,329 | 12.9% | MICE-BR |
| diarrhea | 6,416 | 62.4%* | MICE-BR |
| All other variables | 0 | 0% | N/A |

**Diarrhea only available in 2022 DHS survey; imputed for 2016/2019 using MICE with age, region, and season as predictors. The outcome variable (rdt_test) was excluded from all imputation models to prevent outcome leakage.*

## MICE Configuration

- Estimator: Bayesian Ridge regression
- Imputation method: Bayesian Ridge regression (sklearn IterativeImputer)
- Number of imputations (m): 5
- Maximum iterations: 50
- Convergence tolerance: 0.001
- Predictor variables: age (months), region, season
- **Outcome variable (rdt_test) excluded from imputation model to prevent leakage**

# A.4 Symptom Simulation Parameters

Seven symptom variables not captured in DHS/MIS surveys were synthetically generated using logistic regression models calibrated to literature-derived prevalence targets. The outcome variable (rdt_test) was used only as a grouping variable to parameterise two population-level Bernoulli distributions — one for malaria-positive children and one for malaria-negative children. Each individual child's synthetic symptom value is a stochastic binary draw from the appropriate distribution, not a deterministic transformation of their RDT result. Binary symptom probability: P(symptom=1) = σ(β₀ + β₁·malaria + β₂·fever + ε), where ε ~ N(0, σ²).

**Table A.4: Symptom Simulation Parameters (Binary Symptoms) — Target and Empirical Prevalences**

| **Symptom** | **β₀** | **β₁ (malaria)** | **β₂ (fever)** | **Noise SD** | **Prev. M+** | **Prev. M−** |
| --- | --- | --- | --- | --- | --- | --- |
| Chills | −1.73 | 2.30 | 1.50 | 0.5 | 65% | 15% |
| Sweating | −1.99 | 2.08 | 1.39 | 0.5 | 55% | 12% |
| Nausea/vomiting | −2.20 | 1.79 | 1.10 | 0.4 | 40% | 10% |
| Appetite loss | −1.52 | 1.95 | 1.20 | 0.5 | 58% | 18% |
| Recent travel | −2.44 | 1.10 | 0.00 | 0.6 | 20% | 8% |

*M+/M− = malaria positive/negative. Prev. M+/M− columns show target prevalences derived from published epidemiological literature (Luxemburger et al. 1998; Tangpukdee et al. 2009; WHO Guidelines 2023). Empirical prevalences observed in the simulated dataset are: chills M+ 65.4%/M− 27.4%; sweating M+ 53.5%/M− 22.8%; nausea/vomiting M+ 35.4%/M− 16.7%; appetite loss M+ 48.5%/M− 23.5%; recent travel M+ 14.1%/M− 9.2%. Empirical M− prevalences for chills and sweating substantially exceed targets due to the fever co-predictor in the logistic generation function increasing false-positive symptom rates; this is a generation artefact rather than circularity, as parameters were derived exclusively from independent literature and not from statistical relationships within the DHS dataset.*

**Table A.5: Severity Score Distributions (Ordinal Symptoms)**

| **Severity Level** | **Headache M+** | **Headache M−** | **Bodyaches M+** | **Bodyaches M−** |
| --- | --- | --- | --- | --- |
| 0 (None) | 8% | 55% | 10% | 52% |
| 1 (Mild) | 22% | 30% | 25% | 32% |
| 2 (Moderate) | 42% | 12% | 40% | 13% |
| 3 (Severe) | 28% | 3% | 25% | 3% |

## Validation Results

Chi-square tests confirmed all simulated symptoms significantly associated with malaria status (p < 0.001). Correlation validation: chills–fever ρ = 0.58; headache–bodyaches ρ = 0.62. Empirical positive-class prevalences were within acceptable range for chills (65.4% vs 65% target) and sweating (53.5% vs 55%); nausea/vomiting (35.4% vs 40%), appetite loss (48.5% vs 58%), and recent travel (14.1% vs 20%) showed larger gaps due to fever co-prediction and stochastic noise attenuating the marginal malaria effect. Negative-class prevalences for chills (27.4%) and sweating (22.8%) exceeded targets (15%, 12%) for the same reason. All empirical values are reported in the Table A.4 footnote.

# A.5 Data Splitting and SMOTE Augmentation

**Table A.6: Data Split Summary**

| **Split** | **Samples** | **Positive (%)** | **Purpose** |
| --- | --- | --- | --- |
| Training | 6,172 (60%) | 25.3% | Model training |
| Validation | 2,057 (20%) | 25.3% | Hyperparameter tuning |
| Test | 2,058 (20%) | 25.3% | Final evaluation |

## SMOTE Configuration

**Algorithm:** SMOTENC (handles mixed categorical and continuous features)

**Target positive class ratio:** 35%

**k-neighbors:** min(5, minority samples − 1)

**Centralized training:** SMOTENC applied globally to augment the positive class (1,559 → 2,520; +961 synthetic positives), followed by RandomOverSampling of the majority class (4,613 → 4,680; +67 samples) to reach the exact 7,200-sample target at 35% positive ratio. Final composition: 2,520 positive + 4,680 negative = 7,200 total. The 67 additional negative samples (1.5% majority class increase) were necessary to achieve the exact target size and do not materially alter class balance.

**Federated training:** SMOTE applied locally at each client using only that client's own data. Applied independently per client to prevent cross-client leakage and maintain the privacy guarantee of the federated setting.

# A.6 Sensitivity Analysis: Native DHS Features vs. Full Feature Set

To address concerns about the predictive contribution of synthetically generated symptom variables, a sensitivity analysis was conducted comparing model performance using only the five DHS-native features against the full 12-feature set across all three experimental scenarios, using 10 random seeds (42–51) and the same hyperparameters as the main experiments.

**Native DHS features (5):** fever, diarrhea, bednet_use, season, age_group

**Synthetic features (7):** chills, sweating, headache, bodyaches, nausea_vomiting, appetite_loss, recent_travel

**Table A.7: Sensitivity Analysis Results — AUC-PR (mean ± SD, 10 seeds)**

| **Scenario** | **Algorithm** | **Native-5 AUC-PR** | **Full-12 AUC-PR** | **Δ AUC-PR** |
| --- | --- | --- | --- | --- |
| S1: IID Baseline | FedAvg | 0.3692 ± 0.0024 | 0.8853 ± 0.0004 | +0.5161 |
| S1: IID Baseline | FedProx | 0.3697 ± 0.0013 | 0.8840 ± 0.0017 | +0.5143 |
| S2: Regional Heterogeneity | FedAvg | 0.3412 ± 0.0056 | 0.8679 ± 0.0026 | +0.5267 |
| S2: Regional Heterogeneity | FedProx | 0.3485 ± 0.0067 | 0.8706 ± 0.0023 | +0.5221 |
| S3: Quality Variation | FedAvg | 0.3455 ± 0.0041 | 0.8703 ± 0.0027 | +0.5248 |
| S3: Quality Variation | FedProx | 0.3478 ± 0.0059 | 0.8734 ± 0.0018 | +0.5256 |
| Centralized LR | — | 0.3699 | 0.8854 | +0.5155 |

*Mean Δ AUC-PR across all federated conditions: +0.52 (range: +0.51 to +0.53). FedProx μ = 0.5 throughout. Centralized LR: single run, C = 1.0, balanced class weights. Full-12 AUC-PR column highlighted in green.*

## Interpretation

The mean AUC-PR gain from synthetic features is +0.52 across all scenarios. This large delta reflects the fundamental difference between population-level household survey data and point-of-care clinical assessment: DHS instruments do not capture chills, sweating, headache, bodyaches, nausea, appetite loss, or recent travel by design, whereas these are standard components of a malaria clinical consultation. The native DHS features have weak individual correlations with malaria status (r = 0.05–0.15), consistent with the known non-specificity of two-week household fever recall in Ghanaian children.

Critically, the centralized baseline shows an identical delta (Δ AUC-PR = +0.5155), confirming that the performance gap is driven entirely by the difference between DHS survey features and clinical symptom features — not by any artefact of the federated training mechanism. This is a data availability limitation, not a validity flaw in the evaluation methodology.

The published benchmarks therefore represent expected performance when a clinician records the complete standard symptom panel at point-of-care consultation, which is the intended deployment scenario. Prospective validation with facility-collected clinical records — where these symptoms would be directly measured — is a prerequisite for deployment and is identified as the primary future direction.
