# Supplementary material for: Multi-scenario evaluation of federated learning for privacy-preserving malaria prediction with Ghana DHS data
Source: PLOS Digit Health. 2026 Jul 24;5(7):e0001581. doi: 10.1371/journal.pdig.0001581 (PMC13399501; doi:10.1371/journal.pdig.0001581)
Supplement: S2 Text — Client configurations, three scenarios (IID, regional heterogeneity, quality variation), and FL algorithm pseudocode. (DOCX) [file pdig.0001581.s002.docx]

**S2 Text. Experimental Scenario Specifications**

Detailed client-level data partitioning schemes for IID, non-IID regional, and heterogeneous quality scenarios with sample sizes, prevalence rates, and missing data patterns for federated learning evaluation.

**Table B.1: Experimental Scenario Specification by Client**

| **Scenario** | **Client** | **Region** | **Samples** | **Prevalence** | **Missing %** |
| --- | --- | --- | --- | --- | --- |
| S1: IID | 0 | Random | 1,440 | 25.6% | 0% |
|  | 1 | Random | 1,440 | 24.8% | 0% |
|  | 2 | Random | 1,440 | 25.4% | 0% |
|  | 3 | Random | 1,440 | 26.3% | 0% |
|  | 4 | Random | 1,440 | 24.2% | 0% |
| S2: Non-IID | 0 | Greater Accra | 1,800 | 2.9% | 0% |
|  | 1 | Ashanti | 1,440 | 14.0% | 0% |
|  | 2 | Northern | 1,440 | 30.1% | 0% |
|  | 3 | Upper East | 1,260 | 26.0% | 0% |
|  | 4 | Western | 1,260 | 26.4% | 0% |
| S3: Quality | 0 | Greater Accra | 1,800 | 2.9% | 5% |
|  | 1 | Ashanti | 1,440 | 14.0% | 10% |
|  | 2 | Northern | 1,440 | 30.1% | 20% |
|  | 3 | Upper East | 1,260 | 26.0% | 15% |
|  | 4 | Western | 1,260 | 26.4% | 12% |

*Sample sizes shown are post-SMOTE augmentation. S1 uses stratified partitioning with 35% positive target. S2–S3 use local SMOTE per client, preserving regional prevalence ratios. Missing data in S3 is MCAR (Missing Completely At Random) applied to symptom features only, excluding the outcome variable.*

# B.1 Scenario Descriptions

## S1: IID (Independent and Identically Distributed)

**Purpose:** Establish an artificial upper-bound baseline for federated learning performance under ideal conditions. This scenario is not intended to represent real-world epidemiological conditions but to provide a controlled reference against which heterogeneity effects can be quantified.

**Characteristics:** Random stratified partitioning across 5 clients; uniform sample sizes (1,440 per client); balanced malaria prevalence (~25% across all clients); no missing data; no regional bias.

**Expected behaviour:** FL should match centralized performance with minimal degradation, establishing performance equivalence under homogeneous conditions.

## S2: Non-IID Regional Heterogeneity

**Purpose:** Evaluate FL under naturally occurring epidemiological heterogeneity reflecting Ghana's actual malaria burden distribution.

**Characteristics:** Region-based client assignment based on actual DHS/MIS geographic data; variable sample sizes (1,260–1,800 per client); 10-fold prevalence variation (2.9% in Greater Accra to 30.1% in Northern region); no missing data. Prevalence values are empirically derived from the merged DHS/MIS dataset consistent with the Ghana National Malaria Strategic Plan 2021–2025. This is not an artificially constructed distribution.

**Regional context:**

- **Greater Accra (Client 0):** Urban, coastal, low transmission (2.9%)
- **Ashanti (Client 1):** Mixed urban/rural, moderate transmission (14.0%)
- **Northern (Client 2):** Rural, high transmission zone (30.1%)
- **Upper East (Client 3):** Rural, high transmission (26.0%)
- **Western (Client 4):** Mixed terrain, high transmission (26.4%)

## S3: Heterogeneous Data Quality

**Purpose:** Evaluate FL robustness to variable data completeness across sites, simulating real-world scenarios where some facilities have incomplete records.

**Characteristics:** Inherits regional heterogeneity from S2; additional challenge of variable missing data rates (5–20% per client); missing data applied via MCAR (Missing Completely At Random) mechanism by randomly selecting feature matrix cells (excluding outcome variable and region identifiers) and setting to NaN. MCAR was chosen as the most conservative assumption: if FL is robust under MCAR, it provides a lower bound on robustness under more structured missingness patterns (MAR/MNAR).

**Missing data rates by client:**

- **Client 0 — Greater Accra:** 5% missing (well-resourced urban facility)
- **Client 1 — Ashanti:** 10% missing (regional hospital)
- **Client 2 — Northern:** 20% missing (resource-limited rural facility)
- **Client 3 — Upper East:** 15% missing (rural health centre)
- **Client 4 — Western:** 12% missing (mixed facility)

# B.2 Data Distribution Analysis

**Table B.2: Client Sample Size Distribution**

| **Metric** | **S1 (IID)** | **S2 (Non-IID)** | **S3 (Quality)** |
| --- | --- | --- | --- |
| Min samples | 1,440 | 1,260 | 1,260 |
| Max samples | 1,440 | 1,800 | 1,800 |
| Mean samples | 1,440 | 1,440 | 1,440 |
| Std Dev | 0 | 194.9 | 194.9 |

**Table B.3: Prevalence Distribution Across Clients**

| **Metric** | **S1** | **S2** | **S3** |
| --- | --- | --- | --- |
| Min prevalence | 24.2% | 2.9% | 2.9% |
| Max prevalence | 26.3% | 30.1% | 30.1% |
| Mean prevalence | 25.3% | 19.9% | 19.9% |
| Std Dev | 0.8% | 11.2% | 11.2% |
| Coefficient of variation | 3.2% | 56.3% | 56.3% |

# B.3 Federated Learning Algorithm Pseudocode

## Algorithm 1: Federated Training Procedure (FedAvg / FedProx)

**Inputs:**

K = number of clients

Dk = local training dataset at client k

R = number of communication rounds

E = number of local training epochs per round

η = learning rate (SGD optimizer)

μ = proximal coefficient (μ = 0 → FedAvg; μ > 0 → FedProx)

b = mini-batch size

nk = number of training samples at client k

n = total samples,

n = Σk nk

Output

Trained global model ωR

Server Procedure

Step1. Initialize global ω0 (Xavier unform weighs, zero bias)

Step 2. For t = 1 to R do

Broadcast ωt to all K clients

For each client k ∈ {1, …, K} in parallel do:

D̃k ← LocalSMOTE (Dk) // local augmentation only, no cross-client leakage

ωk ← LocalUpdate (ωt, D̃k, E, η, μ)

end for

Aggregate via FedAvg weighted average:

$$\omega^{t+1}=\sum_{k} \frac{n_{k}}{n}.\omega_{k}$$

(Equation1: FedAvg aggregation)

End for

Step 3. Return ωR

Client Subroutine 1: LocalSMOTE (Dk)

If minority class ratio < 0.35 then

Compute adaptive k-neighbours:

$k_{nn}=min(5,n_{minority}-1)$

(Equation 2: adaptive k-neighbours)

D̃k ← SMOTE (Dk, target_ratio = 0.35, k = knn)

Else

D̃k ← Dk // already balanced

End if

Return D̃k

Client Subroutine 2: LocalUpdate (ωt, D̃k, E, η, μ)

ω ← ωt // initialize from global parameters

for e = 1 to E do:

for each mini-batch B ⊆ D̃k do:

Compute FedProx objective (μ = 0 reduces to BCE — FedAvg):

$$L_{FedProx}\left( \omega\right)=L\left( \omega;B \right)+\frac{\mu}{2}\parallel\omega-\omega^{t}\parallel^{2}$$

$$(Equation 3:FedProx Objective)$$

Apply SGD gradient update:

$\omega⟵\omega-\eta. \nabla_{\omega}L_{FedProx}(\omega)$

$$(Equation 4:SGD parameter update)$$

End for

End for

Return ω

**Notes:**

1. When μ = 0, the proximal term in Eq. 3 vanishes, reducing the objective to standard BCE loss (FedAvg).

2. LocalSMOTE runs independently per client using only local data, preserving the privacy guarantee of the federated setting.

3. Eq. 1 is the FedAvg aggregation rule (McMahan et al., 2017); client contribution is proportional to local dataset size nk.

4. Experimental settings: R = 10, E = 10, η = 0.05, b = 32, K = 5, base seed = 42, repeated over 10 seeds (42–51).

5. FedAvg and FedProx were implemented in PyTorch rather than a dedicated FL framework such as Flower, to maximize transparency and reflect lightweight infrastructure constraints typical of sub-Saharan African health systems.
