# Supplementary material for: Multi-scenario evaluation of federated learning for privacy-preserving malaria prediction with Ghana DHS data
Source: PLOS Digit Health. 2026 Jul 24;5(7):e0001581. doi: 10.1371/journal.pdig.0001581 (PMC13399501; doi:10.1371/journal.pdig.0001581)
Supplement: S3 Text — Grid search configurations, optimization details, convergence criteria, and 600 experiment run specifications. (DOCX) [file pdig.0001581.s003.docx]

**S3 Text. Hyperparameter Settings**

Complete grid search configurations, parameter ranges, and optimization settings for both centralized baseline models and federated learning algorithms used in malaria prediction study.

# **C.1 Centralized Model Hyperparameters**

## **Table C.1: Centralized Model Configuration**

| **Parameter** | **Logistic Regression** | **Random Forest** |
| --- | --- | --- |
| Implementation | sklearn.LogisticRegression | sklearn.RandomForestClassifier |
| Regularization | L2 (Ridge) | N/A |
| Solver/Criterion | LBFGS | Gini impurity |
| Class weights | Balanced | Balanced |
| CV folds | 5 (stratified) | 5 (stratified) |
| Scoring metric | AUC | AUC |

## **Table C.2: Grid Search Ranges – Centralized Models**

| **Model** | **Parameter** | **Search Range** | **Grid Points** |
| --- | --- | --- | --- |
| Logistic Regression | C (regularization) | {0.001, 0.01, 0.1, 1.0, 10.0, 100.0} | 6 |
| Random Forest | n_estimators | {100, 200} | 2 |
| Random Forest | max_depth | {10, 20, None} | 3 |
| Random Forest | min_samples_split | {2, 5} | 2 |
| Random Forest | min_samples_leaf | {1, 2} | 2 |

Total configurations: Logistic Regression – 6; Random Forest – 24 (2 × 3 × 2 × 2). Selection method: 5-fold stratified cross-validation with AUC scoring.

# **C.2 Federated Learning Hyperparameters**

## **Table C.3: Federated Learning Base Configuration**

| **Parameter** | **Value** |
| --- | --- |
| Model architecture | Logistic Regression (12→1) |
| Optimizer | SGD (no momentum) |
| Loss function | Binary cross-entropy |
| Class weighting | n_negative / n_positive |
| Batch size | 32 |
| Communication rounds (R) | 10 |
| Local epochs (E) – baseline | 10 |
| Learning rate (η) – baseline | 0.05 |
| Proximal coefficient (μ) – baseline | 0.1* (FedProx), 0.0 (FedAvg) |
| Weight initialization | Xavier uniform |
| Bias initialization | Zero |
| Feature standardization | Z-score (fit on training) |
| Aggregation weighting | Sample size proportional |

**Final comparison uses μ = 0.5 for FedProx based on ablation study results showing optimal performance under heterogeneous conditions.*

## **Table C.4: Grid Search Configuration**

| **Parameter** | **Search Range** | **Grid Points** |
| --- | --- | --- |
| Proximal coefficient (μ) | {0.0, 0.01, 0.1, 0.5, 1.0} | 5 |
| Learning rate (η) | {0.01, 0.05} | 2 |
| Local epochs (E) | {5, 10, 15} | 3 |
| Random seeds | 42–51 | 10 |
| Algorithms | FedAvg, FedProx | 2 |

Total configurations: 5 × 2 × 3 × 2 algorithms = 60. Total runs: 60 × 10 seeds = 600 experiment runs.

# **C.3 Hyperparameter Selection Rationale**

## **Communication Rounds (R = 10)**

Convergence analysis showed plateau after 6–8 rounds; R = 10 was set to ensure complete convergence across all scenarios. Evidence: S1 (IID) converged by round 5; S2 (Non-IID) by round 6; S3 (Quality) by round 7 (all means).

## **Local Epochs (E)**

Grid search range: {5, 10, 15}. E = 5 led to underfitting; E = 10 was optimal, balancing local training and communication efficiency; E = 15 showed marginal performance gain with increased cost. Selected: E = 10.

## **Learning Rate (η)**

Grid search range: {0.01, 0.05}. η = 0.01 produced stable but slow convergence; η = 0.05 was optimal with faster convergence and acceptable stability; higher rates (0.1, 0.2) were unstable. Selected: η = 0.05.

## **Proximal Coefficient (μ) – FedProx Only**

Grid search range: {0.0, 0.01, 0.1, 0.5, 1.0}. μ = 0.0 reduces to FedAvg; μ = 0.01 minimal effect; μ = 0.1 moderate baseline; μ = 0.5 optimal for heterogeneous scenarios (S2, S3); μ = 1.0 over-regularized. Selected for final comparison: μ = 0.5.

## **Batch Size**

Fixed at 32. Smaller batches (8, 16) produced noisy gradients; batch 32 optimally balanced gradient quality and memory efficiency; larger batches (64, 128) were insufficient given sample sizes per client.

## **Class Weighting**

Method: n_negative / n_positive (inverse class frequency). Without weighting: biased toward majority class (77% accuracy, 30% recall). With weighting: balanced performance (85% accuracy, 87% recall).

# **C.4 Optimization Details**

## **Loss Function**

Binary Cross-Entropy with Class Weights: L(ω; D) = –(1/n) Σᵢ [wᵢ · yᵢ log(ŷᵢ) + (1–yᵢ) log(1–ŷᵢ)], where wᵢ = n_negative/n_positive if yᵢ = 1, and wᵢ = 1 if yᵢ = 0.

## **FedProx Proximal Term**

Regularized objective: min_ω L(ω; Dₖ) + (μ/2)||ω – ωᵗ||². Higher μ → slower local convergence but better global stability; constrains local updates to stay close to global model and reduces client drift under heterogeneity.

## **Aggregation Strategy**

Sample-size weighted averaging: ωᵗ⁺¹ = Σₖ (nₖ/n) · ωₖ, where nₖ = number of samples at client k and n = total samples. Clients with more data have proportionally greater influence on the global model.

# **C.5 Convergence Criteria**

Training stops when: (1) maximum rounds reached (R = 10); (2) validation loss plateau – no improvement for 3 consecutive rounds; or (3) validation AUC plateau – ΔAUC < 0.001 for 3 consecutive rounds. Early stopping was not used to ensure fair comparison across all scenarios.

Primary metric: AUC-PR (Area Under Precision-Recall Curve). Secondary metrics: AUC-ROC, Accuracy, Precision, Recall, F1-Score. Communication efficiency measured as number of rounds to convergence.

# **C.6 Statistical Validation**

## **Multiple Seeds**

Seeds used: 42–51 (n = 10). Purpose: quantify performance variance, ensure results are not due to random initialization, and provide confidence intervals for metrics.

## **Statistical Tests**

DeLong's test for AUC comparisons (paired); Bootstrap CI with 1,000 iterations and 95% confidence intervals; paired t-test for cross-scenario comparisons; Cohen's d for effect size quantification.

# **C.7 Computational Resource Requirements**

## **Per Experiment Run**

Training time: S1 (IID) ~12 minutes; S2 (Non-IID) ~14 minutes; S3 (Quality) ~16 minutes.

Total computational cost: 600 runs × ~14 min average = 140 hours (5.8 days). Parallelization: 4 runs simultaneously → 35 hours actual time.

Memory: Peak RAM ~2.3 GB per run; Disk ~450 MB logs per run → ~270 GB total.

Hardware: CPU 95–100% (single core per run); GPU not used (reproducibility constraint).
