# Supplementary material for: Multi-scenario evaluation of federated learning for privacy-preserving malaria prediction with Ghana DHS data
Source: PLOS Digit Health. 2026 Jul 24;5(7):e0001581. doi: 10.1371/journal.pdig.0001581 (PMC13399501; doi:10.1371/journal.pdig.0001581)
Supplement: S4 Text — Software versions, random seed protocols, experiment logging, and code repository details. (DOCX) [file pdig.0001581.s004.docx]

**S4 Text. Reproducibility Details**

Software environment specifications, random seed protocols, experiment logging procedures, repository structure, and code availability details for complete reproducibility of the federated learning malaria prediction study.

# D.1 Software Environment

**Table D.1: Software Versions**

| **Package** | **Version** | **Purpose** |
| --- | --- | --- |
| Python | 3.10 | Programming language |
| PyTorch | 2.9.1 | Federated model training |
| scikit-learn | 1.7.2 | Centralized baselines, metrics |
| pandas | 2.3.3 | Data manipulation |
| numpy | 2.1.3 | Numerical computing |
| imbalanced-learn | 0.14.1 | SMOTENC implementation |
| statsmodels | 0.14.5 | Statistical testing |
| scipy | 1.11 | Scientific computing |
| matplotlib | 3.10.6 | Visualization |
| seaborn | 0.13.2 | Statistical visualization |
| pillow | 11.3.0 | Image processing |
| joblib | 1.5.2 | Serialization |
| tqdm | 4.67.1 | Progress bars |
| PyYAML | 6.0.3 | Configuration files |

## System Configuration

- Operating System: Windows 11 (64-bit)
- CPU: AMD Ryzen 5 PRO 4650U (2.10 GHz, 6 cores / 12 threads)
- RAM: 16 GB DDR4 | Storage: 512 GB SSD
- Training device: CPU only — ensures deterministic reproducibility across hardware configurations

# D.2 Random Seed Protocol

All random operations were seeded deterministically. Base seed: 42. Run-specific seed: 42 + run_id (seeds 42–51 for runs 0–9). Data split seed fixed at 42 across all experiments so all algorithms are trained and evaluated on identical data partitions.

def set_seed(seed):

random.seed(seed)

np.random.seed(seed)

torch.manual_seed(seed)

torch.cuda.manual_seed_all(seed)

torch.backends.cudnn.deterministic = True

torch.backends.cudnn.benchmark = False

# D.3 Repository Structure

The repository is organized to separate data extraction, preprocessing, federated training, evaluation, and configuration. All scripts are in src/training/; raw and processed data remain local per DHS data use terms.

***Table D.2: Repository File Structure and Description***

| **Path** | **File / Directory** | **Description** |
| --- | --- | --- |
| FL_malaria/ |  | Project root |
|  | **README.md** | Pipeline walkthrough and quick-start guide |
|  | **requirements.txt** | Pip dependencies with pinned versions |
|  | **environment.yml** | Conda environment specification |
|  | **run_experiments.py** | Main experiment runner — reproduces all results |
|  | Dockerfile | Docker environment for complete platform replication |
| data/ |  | Data directories (not committed; reproduce via pipeline) |
| data/ | raw/ | Raw DHS/MIS .DTA Stata files from dhsprogram.com |
| data/ | merged/ | Merged dataset (ghana_malaria_merged.csv) |
| data/ | cleaned/ | Preprocessed train / val / test splits |
| data/cleaned/ | **train_centralized.csv** | SMOTE-augmented centralized training set (n=7,200) |
| data/cleaned/ | **val_set.csv** | Validation set — original, no SMOTE (n=2,058) |
| data/cleaned/ | **test_set.csv** | Held-out test set — original, no SMOTE (n=2,057) |
| data/ | fl_scenarios/ | Federated client partition root |
| data/fl_scenarios/ | s1_iid/ | S1 IID — client_0.csv … client_4.csv |
| data/fl_scenarios/ | s2_noniid/ | S2 Regional heterogeneity — client_0.csv … client_4.csv |
| data/fl_scenarios/ | s3_quality/ | S3 Quality variation — client_0.csv … client_4.csv |
| src/training/ |  | All executable scripts |
| src/training/ | **data_extraction.py** | DHS/MIS Stata file extraction and dataset merging |
| src/training/ | **data_preprocessing.py** | MICE imputation, symptom simulation, SMOTE, splits |
| src/training/ | **create_fl_scenarios.py** | Federated client partition generation (S1/S2/S3) |
| src/training/ | **train_centralized.py** | Centralized LR and RF with GridSearchCV (AUC-PR) |
| src/training/ | **train_federated.py** | FedAvg and FedProx training loop (10 seeds) |
| src/training/ | **sensitivity_analysis.py** | Native DHS vs full feature set comparison |
| src/training/ | **final_analysis.py** | Results aggregation, statistical tests, figures |
| results/ |  | Generated outputs (created at runtime) |
| results/ | **centralized_results.json** | Centralized model metrics and predictions |
| results/ | **sensitivity_analysis.json** | Sensitivity analysis full results |
| results/ | fedavg/ | FedAvg results by scenario (JSON per seed per scenario) |
| results/ | fedprox/ | FedProx results by scenario (JSON per seed per scenario) |
| results/ | figures/ | Generated figures (Fig 1–6, .png and .tif) |
| validation/ |  | Data quality verification outputs |
| validation/ | **yearly_weighted_prevalence.csv** | Prevalence by survey year |
| validation/ | **regional_weighted_prevalence.csv** | Prevalence by region — used in S2/S3 |
| src/config/ | **scenarios.yaml** | Scenario definitions and client specifications |
| src/config/ | **hyperparams.yaml** | Hyperparameter grid and training settings |
| tests/ | **test_preprocessing.py** | Data pipeline unit tests |
| tests/ | **test_federated.py** | FL algorithm unit tests |
| tests/integration/ | **test_pipeline.py** | End-to-end pipeline integration test |

# D.4 Pipeline Execution Order

Scripts must be run in the following order from the project root directory. Steps 1–3 require DHS data access; steps 4–7 require only the cleaned data files.

***Table D.3: Pipeline Execution Sequence***

| **Step** | **Script** | **Purpose** | **Output** |
| --- | --- | --- | --- |
| 1 | data_extraction.py | Extract and merge DHS/MIS Stata files | data/merged/ghana_malaria_merged.csv |
| 2 | data_preprocessing.py | MICE imputation, symptom simulation, SMOTE, train/val/test split | data/cleaned/*.csv |
| 3 | create_fl_scenarios.py | Generate federated client partitions for S1, S2, S3 | data/fl_scenarios/**/*.csv |
| 4 | train_centralized.py | Centralized LR and RF with 5-fold GridSearchCV | results/centralized_results.json |
| 5 | train_federated.py | FedAvg and FedProx across all scenarios, 10 seeds | results/fedavg/ and results/fedprox/ |
| 6 | sensitivity_analysis.py | Native DHS vs full feature set comparison | results/sensitivity_analysis.json |
| 7 | final_analysis.py | Statistical tests, figures, summary tables | results/figures/ and results/tables/ |

## Quick Start — Reproduce All Results

git clone https://github.com/danielke32/FL_malaria.git

cd FL_malaria

pip install -r requirements.txt

python run_experiments.py --all --seeds 42-51

## Run Individual Steps

# Step 4: centralized baseline only

python src/training/train_centralized.py

# Step 5: federated, specific scenario and algorithm

python src/training/train_federated.py --scenario s2_noniid --algorithm fedavg --seed 42

# Step 6: sensitivity analysis

python src/training/sensitivity_analysis.py

# D.5 Code Availability

**Primary repository:** https://github.com/danielke32/FL_malaria

**License:** MIT License (open source, public)

**Archived version (Zenodo):** https://doi.org/10.5281/zenodo.18138045

**Archive contents:** Complete codebase at time of submission including requirements.txt, README.md, and all pipeline scripts

# D.6 Data Availability

Raw DHS/MIS data are available from The DHS Program at https://dhsprogram.com/ (public access, registration required). Processed data are not committed to the repository per DHS data use agreement, but are fully reproducible via steps 1–3 of the pipeline above.

**Table D.4: Datasets Used**

| **Dataset** | **Year** | **Type** | **DHS File Reference** |
| --- | --- | --- | --- |
| Ghana Malaria Indicator Survey | 2016 | MIS | GHPR7BFL.DTA / GHKR7BFL.DTA |
| Ghana Malaria Indicator Survey | 2019 | MIS | GHPR82FL.DTA / GHKR82FL.DTA |
| Ghana Demographic and Health Survey | 2022 | DHS | GHPR8CFL.DTA / GHKR8CFL.DTA |

# D.7 Docker Support

A Dockerfile is included for complete environment replication across Linux, Windows, and macOS.

# Build and run

docker build -t fl_malaria .

docker run -v $(pwd)/results:/app/results fl_malaria --all

# D.8 Verification

## Unit Tests

**pytest tests/ -v --cov** Coverage: data preprocessing, SMOTE, FL algorithms, evaluation metrics, statistical tests.

## Integration Tests

Location: tests/integration/. Tests: end-to-end training pipeline; multi-scenario execution; results consistency across seeds.

## Validation Outputs

- **validation/yearly_weighted_prevalence.csv:** Verify prevalence matches published DHS reports
- **validation/regional_weighted_prevalence.csv:** Confirm regional prevalence values used in S2/S3

# D.9 Citation

@article{kovor2026federated,

title = {Multi-scenario evaluation of federated learning for

privacy-preserving malaria prediction with Ghana DHS data},

author = {Kovor, Daniel Kwasi and Osei, Eric Opoku},

journal = {PLOS Digital Health},

year = {2026},

doi = {10.1371/journal.pdig.XXXXXXX}

}

# D.10 Support and Contact

**GitHub Issues:** https://github.com/danielke32/FL_malaria/issues

**Corresponding author:** Daniel Kwasi Kovor — dkkovor@st.knust.edu.gh

**Institution:** Department of Computer Science, Kwame Nkrumah University of Science and Technology, Ghana
